# Abundance, distribution, and expression of nematicidal crystal protein genes in *Bacillus thuringiensis* strains from diverse habitats

**DOI:** 10.1007/s10123-022-00307-z

**Published:** 2022-12-09

**Authors:** Yolanda Bel, Miguel Andrés-Antón, Baltasar Escriche

**Affiliations:** Institut de Biotecnologia i Biomedicina (BIOTECMED), Departament de Genètica, Universitat de València, C/Dr. Moliner, 50, 46100 Burjassot, Spain

**Keywords:** Nematode, Cry proteins, *Cry* genes, Bt toxins, PCR screening, Proteomics

## Abstract

*Bacillus thuringiensis* (Bt) is a Gram-positive bacterium that accumulates pesticidal proteins (Cry and Cyt) in parasporal crystals. Proteins from the Cry5, App6 (formerly Cry6), Cry12, Cry13, Cry14, Cry21, and Xpp55 (formerly Cry55) families have been identified as toxic to nematodes. In this study, a total of 846 Bt strains belonging to four collections were analyzed to determine the diversity and distribution of the Bt Cry nematicidal protein genes. We analyzed their presence by PCR, and positives were confirmed by sequencing. As a result, 164 Bt isolates (20%) contained at least one gene coding for nematicidal Cry proteins. The *cry5* and *cry21* genes were enriched in collection 1 and were often found together in the same strain. Differently, in collection 4, obtained from similar habitats but after 10 years, *cry14* was the gene most frequently found. In collection 2, *cry5* and *app6* were the most abundant genes, and collection 3 had a low incidence of any of these genes. The results point to high variability in the frequencies of the studied genes depending on the timing, geographical origins, and sources. The occurrence of *cry1A*, *cry2*, and *cry3* genes was also analyzed and showed that the nematicidal Cry protein genes were frequently accompanied by *cry1A* + *cry2*. The expression of the genes was assessed by mass spectrometry showing that only 14% of the positive strains produced nematicidal proteins. To our knowledge, this is the first comprehensive screening that examines the presence and expression of genes from the seven known Bt Cry nematicidal families.

## Introduction

*Bacillus thuringiensis* (Bt) is an aerobic, spore-forming, Gram-positive bacterium characterized by the production of pesticidal proteins. Among these, the most known are Cry proteins, which accumulate in parasporal crystal inclusion bodies along the sporulation phase (Palma et al. 2014; Gonzalez-Vazquez et al. 2021). Cry proteins are highly toxic to a wide variety of insects, nematodes, mites, protozoa, and human cancer cells (Schnepf et al. 1998; Jouzani et al. 2017; Palma et al. 2014; van Frankenhuyzen 2009; Ohba et al. 2009). Its specificity and lack of toxicity to vertebrates (WHO-IPCS 1999) have made Bt the most successful bio-insecticide used in the last decades worldwide (Jouzani et al. 2017). Bt has demonstrated its potential and safety as a biocontrol agent representing a clear alternative to chemical insecticides. Indeed, it accounts for about 90% of all biopesticides marketed worldwide (Kumar et al. 2021).

The search for new Bt insecticidal proteins has been associated with Bt since this bacterium was discovered in the early years of the last century (Sanahuja et al. 2011). Screening programs have been carried out all over the world, and numerous Bt collections are maintained nowadays in public and private institutions (e.g., the *Bacillus thuringiensis* collection at the Bacillus Genetic Stock Center, Zeigler 1999, Bel et al. 1997, Bravo et al. 1998, Hernández-Rodríguez and Ferré 2009, Djeane et al. 2017, Boonmee et al. 2019). Their purpose is to maintain them as a reservoir of possible novel pesticidal proteins or a source of protein variants with increased pesticidal activity.

Nematodes are one of the most abundant groups of invertebrates in the world, including free-living forms as well as animal or plant parasites. Its species diversity has been estimated to range between 100,000 and 10 million (Poinar 2011). Animal parasitic nematodes affect human health (Stepek et al. 2006), cause large problems in livestock, and constitute one of the most important factors affecting animal production (Sinott et al. 2012). Additionally, there are about 4100 species of plant-parasitic nematodes described (Jones et al. 2013) that are crop pests throughout the world, causing severe damage to plants and accounting for about 80 (Nicol et al. 2011) to 125 billion dollars in losses annually, 14% of the crop losses worldwide (Mesa-Valle et al. 2020; Chitwood 2003).

It has been shown that some free-living nematode species are susceptible to Bt toxins (Iatsenko et al. 2014; Wei et al. 2003). Also, there are several studies showing that Bt Cry proteins are toxic to animal (Wei et al. 2003; Cappello et al. 2006; Kotze et al. 2005; Urban et al. 2013; Hu et al. 2010) or to plant parasitic nematodes (Li et al. 2007, 2008; Guo et al. 2008; Yu et al. 2015; Wang et al. 2012; Verduzco-Rosas et al. 2021; Peng et al. 2011; Cheng et al. 2018; Chinheya et al. 2017). In the toxicity studies carried out with Cry proteins against nematodes, it has been found that LC_50_ values are low (summarized in Jouzani et al. 2017), which indicates that Bt nematicidal proteins can be very useful for the control of these organisms. Therefore, Bt can be considered a good, safe, and low-cost alternative to chemicals for nematode treatment. In this context, the search and discovery of native Bt strains with nematicidal activity could represent a breakthrough in the control of pest nematodes worldwide.

Seven Bt Cry protein families toxic to nematodes have been identified so far: Cry5, Cry6 (App6 according to the new *Bacillus* pesticidal protein nomenclature Crickmore et al. (2022), Cry12, Cry13, Cry14, Cry21, and Cry55 (Xpp55 according to the new nomenclature) (Palma et al. 2014; Jouzani et al. 2017). The aim of this work was to detect Bt strains containing genes coding for nematicidal proteins belonging to the seven nematicidal Cry protein families, out of a total of about 850 Bt strains divided into four collections, to study the nematicidal protein gene frequencies and combinations in nature, and to know if their presence was linked to the ecosystem source, to the content in other insecticidal *cry* genes and also whether there was a correlation with the parasporal crystal morphology. The work unravels the existence of several Bt strains that may be the source of possible new genes that can be considered as an arsenal for increased or new activities. Additionally, the expression of the genes coding for nematicidal Cry proteins in the positive strains was assessed. The strains that produced nematicidal proteins alone or in combination can be considered optimal candidates to combat pest nematodes with significant socioeconomic and health impacts.

## Materials and methods

### *Bacillus thuringiensis* strains

The 846 Bt strains used in this study belonged to four Bt collections stored in the facilities of the Institut BIOTECMED, in the Department of Genetics of the University of Valencia. On the whole, the Bt strains had been isolated in different time periods starting in 1997 as part of an extensive screening to have a source of potential Bt insecticidal strains and proteins. Collections 1 and 4 were composed of Bt strains extracted from samples collected from diverse habitats in Spain in two different time periods that are 10 years apart (Iriarte et al. 1998; Ferrandis et al. 1999; Hernández-Rodríguez and Ferré 2009). Collection 2 consisted of Bt strains obtained from samples related to olive habitats (Bel et al. 1997), and collection 3 consisted of Bt strains extracted from samples collected from potato tubers and potato habitats in Bolivia (Hernandez et al. 2005). The Bt collections used in this study comprise the Bt strains analyzed formerly for different purposes (Iriarte et al. 1998; Hernández-Rodríguez and Ferré 2009; Bel et al. 1997, Hernández et al. 2005) as well as other Bt strains not included in the previous studies.

The samples from where the Bt strains were extracted consisted of soil, storage houses dust, water, etc., collected in sterile containers. For Bt isolation, the liquid samples were processed directly whereas for solid samples, approx. 1 g of solid material was suspended in 10 ml sterile 0.05 M sodium phosphate buffer pH 7 or sterile distilled water. The sample processing and the Bt isolation process from the samples were performed as described previously (Bel et al. 1997). The Bt strains were stored as glycerinates, at − 20 °C in 50% glycerol and at − 80 °C in 15% glycerol. To culture the Bt strains, Luria Bertani Broth (LB media, MacWilliams and Liao 2006), CCY (Stewart et al. 1981) liquid media, or CCY-agar plates were used.

The Bt BMB171 strain carrying and expressing the *cry5Ba* gene and the strain YBT-1518 (that harbors and expresses the *xpp55Aa1* and *app6Aa2* genes) (Guo et al. 2008; He et al. 2010) were kindly supplied by Dr. Sun (State Key Laboratory of Agricultural Microbiology, Huazhong Agricultural University, Wuhan, People’s Republic of China). The Bt strain DB27 (which contains several *cry21* genes (Iatsenko et al. 2014)) was kindly provided by Dr. Sommer (Max Planck Institute for Developmental Biology, Department of Evolutionary Biology, Tübingen, Germany). The Bt strain NRRL B-18246, harboring the *cry13* gene (Narva et al. 1991; Zeigler 1999), was obtained from the Agricultural Research Service Culture Collection (ARS Culture Collection, NRRL, Peoria, IL, USA). These strains were used as positive PCR controls for *cry5*, *xpp55*, *cry21* and *cry13* genes, respectively.

### *Bacillus thuringiensis* microscopic characterization

The Bt strains were plated on CCY-agar plates and incubated for 2 days at 29 °C to induce sporulation. Sporulated Bt strains were checked for the presence of crystal inclusion bodies by Phase Contrast Microscopy using a NIKON Eclipse E400 microscope, at 100 × . The Bt strains were classified according to the parasporal crystal inclusion shape in the categories of bipyramidal, round, irregular, adhered to the spore, two-crystals, and small crystal.

For scanning electron microscopy (SEM) analysis, the sporulated Bt strains were washed in sterile distilled water 3 times. Then, suspensions were air-dried, coated with Gold/Palladium for 2 min in a Sputtering Polaron SC 7649, and observed with a Hitachi S-4800 Scanning Electron Microscope operated at 10 kV.

### DNA extraction, PCR analyses, and sequencing

Total DNA was extracted from Bt vegetative cells grown in LB medium o/n at 29 °C, 180 rpm. Cultures were treated according to the procedure described by Ferrandis et al. (Ferrandis et al. 1999). For PCR detection of the *cry* genes coding for nematicidal proteins, specific primers previously published in the literature were used, as well as primers designed in this work (using Geneious software, Geneious 10.1.3, Biomatters Ltd., Auckland, New Zealand) in order to broaden the range of detection, taking into account the gene alleles described to date (see Table 1 for primer sequences and details).

**Table 1:** Characteristics of general and specific primers used for detection of *cry5*, *app6*, *cry12*, *cry13*, *cry14*, *cry21*, *xpp55*, *cry1*, *cry2*, and *cry3* genes

Primer pair	Gene detected	Sequence (5′–3′)	Annealing temperature (ºC)		Amplicon size (bp)	Reference
Cry5_F	*cry5*	TAAGCAAAGCGCGTAACCTC	52 ºC	300		Ejiofor and Johnson (2002)
Cry5_R		GCTCCCCTCGATGTCAATG				
Cry5B_F1	*cry5B*	TGCGTTACTACGTATCCGCTGT	58 ºC	1.100		This work
Cry5B_R1		AGCCGTATCAAAACGTGAAGC				
Cry6_F	*app6*	TGGCGTAGAGGCTGTTCAAGTA	60 ºC	400		Ejiofor and Johnson (2002)
Cry6_R		TGTCGAGTTCATCATTAGCAGTGT				
Cry6A_F1	*app6A*	TAAGAAATATGGTCCTGGTGATATGAC	56 ºC	1.000		This work
Cry6A_R1		CATCAGAAGCGTCCTCAAGTT				
Cry12_F	*cry12*	CTCCCCCAACATTCCATCC	60 ºC	400		Ejiofor and Johnson (2002)
Cry12_R		AATTACTTACACGTGCCATACCTG				
Cry12_F1	*cry12*	TGCTTTTCAACAATTAGATACAA	60 ºC	450		This work
Cry12_R1		CGAGTTGCGTTTTTGCTGTT				
Cry13_F	*cry13*	CTTTGATTATTTAGGTTTAGTTCAA	52 ºC	300		Bravo et al. (1998)
Cry13_R		TTGTAGTACAGGCTTGTGATTC				
Cry13_F1	*cry13*	GGAAAAACAATGTCGCA	52 ºC	400		This work
Cry13_R1		CAGCAGAAACAGTCCGTA				
Cry14_F	*cry14*	ATAATGCGCGACCTACTGTTGT	58 ºC	450		Ejiofor and Johnson (2002)
Cry14 _R		TGCCGTTATCGCCGTTATT				
Cry14_F1	*cry14*	CTAGTAGGTGGAAATTTTGAAAC	58 ºC	500		This work
Cry14_R1		TCGATTGACTGCTTGAATTTC				
Cry21_F	*cry21*	AGTCGTTGTGTCTCGCTACG	58 ºC	400		This work
Cry21_R		GGAGTGTTGCCGCTACTTCT				
Cry55_F	*xpp55*	ACTGCTGTACGTTCACCAGA	55 ºC	300		This work
Cry55_R		TGCTATGCTAGATCCAACGAGT				
Cry55_F1	*xpp55*	GCTCAAACGTTCTAGTCCCA	58 ºC	750		This work
Cry55_R1		GTTGGTGTTGGATCAGGTG				
Un1_F	*cry1*	CATGATTCATGCGGCAGATAAAC	50 ºC	300		Ben-Dow et al. (1997)
Un1_R		TTGTGACACTTCTGCTTCCCATT				
Un2_F	*cry2*	GTTATTCTTAATGCAGATGAATGGG	50 ºC	700		Ben-Dow et al. (1997)
Un2_R		CGGATAAAATAATCTGGGAAATAGT				
Un3_F	*cry3*	CGTTATCGCAGAGAGATGACATTAAC	50ºC	700		Ben-Dow et al. (1997)
Un3_R		CATCTGTTGTTTCTGGAGGCAAT				

For the PCR screening, 100 ng of DNA template was added to a mixture of 12.5 µl of the NZYTaq II enzyme (5 U/µl) and 1 µl of each primer (10 µM) in a final volume of 25 µl. Amplifications were carried out in an Eppendorf Mastercycler thermal cycler under the following conditions: an initial denaturation step of 3 min at 95 °C followed by 35 cycles of amplification with 30-s denaturation at 94 °C, 30 s of annealing at a variable temperature depending on the primer pair (see Table 1), and 30-s of extension at 72 °C. An extra extension step of 10 min at 72 °C was added after the completion of 35 cycles. The PCR products were visualized by staining with ethidium bromide after separation in 1% agarose gel electrophoresis in 1% TBE buffer (VWR Chemicals, Solon, Ohio, USA), pH 7.5.

For every positive strain, the amplicon either with expected or unexpected sizes was purified and sequenced. PCR products were purified using the NucleoSpin® Gel and PCR Clean-up kit using the instructions supplied by the manufacturer (Macherey–Nagel, Düren, Germany). Purified samples were sequenced in the facilities of STABVida (Caparica, Portugal). The sequences were analyzed, identified, and pairwise aligned using Geneious Prime 10.1.3 software (http://www.ncbi.nlm.nih.gov/BLAST/https://www.geneious.com).

### Statistical analysis

The expected number of strains with a singular gene combination in the case of gene random occurrence was calculated based on individual proportions. The statistical comparison with the observed number of strains was performed with contingency tables using the Fisher’s exact test (Fisher 1922) using GraphPad Prism version 7.0 for Windows, (GraphPad Software, San Diego, California USA, www.graphpad.com). Figures were considered statistically different when *P*-value < 0.05.

### Proteomic analysis

The protein identification of the solubilized crystals was performed as described by Khorramnejad et al. (2020). In short, the Bt strains were grown in liquid CCY medium for 2 days to allow sporulation. Sporulated cultures were centrifuged at 15,900 × *g*, 20 min at 4 °C to recover spores and crystals, which were washed for 3 times in 1 M NaCl, 10 mM EDTA, and once in 10 mM KCl. After the last washing step, the proteins in the crystals were solubilized by resuspending the pellet in 50 mM carbonate buffer, 10 mM DTT, pH 10.5, and shaking o/n at 4 °C. The sample was then centrifuged at 24,000 × *g* for 15 min at 4 °C, and the supernatant was used for protein detection. The detection of the protein content was performed by LC–MS/MS at the proteomics facility of the SCIE (Servei Central de Suport a la Investigació Experimental) at the University of Valencia as described elsewhere (Khorramnejad et al. 2020), after trypsin digestion using a Mass Spectrometter nanoESI qQTOF (6600plus TripleTOF, ABSCIEX). The Protein Pilot v 5.0 (ABSciex, Madrid, Spain) default parameters were used to generate the peptides list, and the protein identification was obtained by the Paragon algorithm (Shilov et al. 2007), using two different databases: the SwissProt database (versión 200,601) and a customized database kindly provided by Dr. Joaquin Gomis-Cebolla (Polytechnic University of Valencia, Spain) that groups all the Cry, Cyt, Mtx-like, Bin-like, Sip, and Vip proteins available in public databases and patents. The LC–MS/MS identification results with at least 95% confidence were considered significant.

## Results

### Screening of nematicidal Cry protein genes in four collections from diverse habitats

**Fig. 1:**
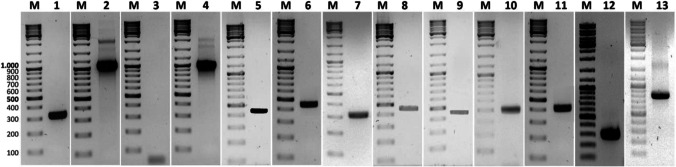
Agarose gel electrophoresis of PCR products obtained using 13 different primer pairs for the detection of nematicidal Cry protein genes. Lane1: V-C5 with primers Cry5_F/R. Lane2: V-C5 with primers Cry5_F_1_/R_1_. Lane3: V-AB6 with primers Cry6_F/R. Lane4: V-AB6 with primers Cry6A_F_1_/R_1_. Lane5: O-V12 with primers Cry12_F/R. Lane6: O-V12 with primers Cry12_F_1_/R_1_. Lane7: NRRL B-18246 with primers Cry13_F/R. Lane8: NRRL B-18246 with primers Cry13_F_1_/R_1_. Lane9: A-V14 with primers Cry14_F/R. Lane10: A-V14 with primers Cry14_F_1_/R_1_. Lane11: N-S21 with primers Cry21_F/R. Lane12: YBT-1518 with primers Cry55_F/R. Lane13: YBT-1518 with primers Cry55_F_1_/R_1_. Lane M: 100 bp DNA ladder (ThermoScientific, Vilnius, Lithuania)

**Fig. 2:**
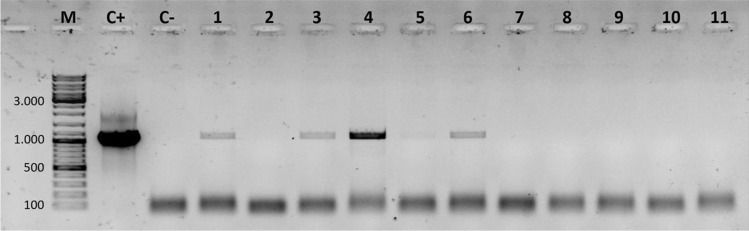
Agarose gel electrophoresis of PCR products obtained using primers Cry5_F_1_/R_1_ for detection of *cry5* gene in 11 field-collected Bt strains randomly selected (lanes 1–11). Lane C + : strain BMB171. Lane C − : negative control (no DNA). Lane M: 100 bp DNA ladder (ThermoScientific, Vilnius, Lithuania)

The screening was based on a PCR system to detect the genes coding for proteins of the Bt Cry families Cry5, App6, Cry12, Cry13, Cry14, Cry21, and Xpp55, using the primer pairs described in Table 1. The primers were either obtained from the bibliography or designed for this work based on specific gene family regions, which is especially important for genes with several known alleles, as is the case of *cry5*, *app6*, or *cry21* genes. Figure 1 shows an example of the PCR products obtained with different wild strains or with standard strains, showing the expected size of the amplicons that depend on the amplified gene and primers. It is worth to notice that for the *app6* gene, no amplicons were obtained when using the Cry6_F/R primers described in the bibliography (Ejiofor and Johnson 2002) which were found to be specific for *app6B* allele. But amplification with Cry6A_F/R primers designed in this work (*app6A* specific) was successful in 2.3% of the Bt isolates. For every positive strain, the amplicon was purified and sequenced to confirm the presence of the gene (as an example, Fig. 2 shows the results of the screening of 11 Bt colonies for *cry5* gene).

The summary of the screening results is shown in Table 2. As can be observed, 164 out of the 846 strains analyzed (20%) carried at least one nematicidal Cry protein gene (Table 2). The individual analysis of each collection showed that this percentage varies from 5% in collection 3 to 33% in collection 1, having nematicidal Cry protein genes 15% of the strains in collections 2 and 4. It is worth noting that *cry13* or *xpp55* genes were not found in any of the collections. In contrast, the four screened collections contained genes coding for Cry5 and Cry21 proteins.

**Table 2:** Amount of Bt strains with nematicidal Cry protein genes detected in total and broken down per collection. The relative amount of positive strains out of the total per collection is indicated between brackets

Genes	Collection 1 (278 strains)	Collection 2 (195 strains)	Collection 3 (110 strains)	Collection 4 (263 strains)	Total (846 strains)
*cry5*	43 (16%)	9 (5%)	2 (2%)	12 (5%)	66 (8%)
*app6*	1 (0.4%)	7 (4%)	0	1 (0.4%)	9 (1%)
*cry12*	0	3 (2%)	1 (1%)	3 (1%)	7 (1%)
*cry13*	0	0	0	0	0
*cry14*	1 (0.4%)	0	1 (1%)	17 (8%)	19 (2%)
*cry21*	18 (7%)	4 (2%)	1 (1%)	4 (2%)	27 (3%)
*xpp55*	0	0	0	0	0
*cry5* + *app6*	3 (1%)	2 (1%)	0	0	5 (1%)
*cry5* + *cry12*	0	1 (0.5%)	0	0	1 (0.1%)
*cry5* + *cry21*	22 (8%)	0	0	0	22 (3%)
*app6* + *cry12*	0	1 (0.5%)	0	0	1 (0.1%)
*app6* + *cry14*	0	1 (0.5%)	0	0	1 (0.1%)
*cry6* + *cry21*	1 (0.4%)	0	0	0	1 (0.1%)
*cry14* + *cry21*	0	0	0	2 (1%)	2 (0.1%)
*cry5* + *app6* + *cry21*	2 (0.7%)	0	0	0	2 (0.2%)
*app6* + *cry14* + *cry21*	0	0	0	1 (0.4%)	1 (0.1%)
At least one gene	91 (33%)	29 (15%)	5 (5%)	40 (15%)	164 (20%)

Based on the proportion of strains with a gene, the expected random gene combinations were determined and statistically compared against the number of observed strains with such combinations. The only value statistically significant was obtained for the combination *cry5* + *cry21* (*P* = 0.003), which pointed out that these two genes were frequently genetically linked since the observed data (22 strains) was higher than expected (6.4). The statistical analyses indicated no genetic linkage for the other gene combinations.

Collections 1 and 4 had been obtained from diverse habitats in Spain, including rural (uncultivated fields, trails, crops, forests) and urban (gardens) surroundings, as well as dust samples from factories, farms, mills, and storehouses. Despite the similarity in the origins of the samples in collection 1 and collection 4, a clear difference was observed in the frequencies with which the different nematicidal Cry protein genes appeared (Table 2). The abundance of genes encoding nematicidal Cry proteins in collection 4 was half of the one observed in collection 1. Additionally, *cry5* and *cry21* were the most abundant genes in collection 1, either alone or combined, but *cry14* was the most abundant gene in collection 4, followed by *cry5*. Besides, *cry 12* was obtained in collection 4 while it was not detected in collection 1.

Collections 2 and 3 were obtained from samples collected from habitats associated with olive tree environments in Spain and potato-growing areas in Bolivia respectively. Although the samples of collection 3, differently to the rest of samples, did not come from Spain, they were included in this work since it was considered interesting for this study to analyze another specific cultivation habitat apart from that of the olive tree–related samples. Collection 3 resulted poor in strains with nematicidal Cry protein genes as only 5 out of 110 carried just one of these genes (1 coming from a storehouse facility, 1 from a factory, 2 from the soil of potato fields, and 1 from non-cultivated countryside soil). Collection 2 resulted richer in Bt strains with genes encoding nematicidal Cry proteins (15% of positive strains), with *cry5* and *app6* as the most abundant genes, being olive tree orchards the main origin of the positive samples. In general, the ecosystems with the highest frequency of Bt strains with nematicidal Cry protein genes were storehouses, mills, and agricultural soils (Table 3).

**Table 3:** Distribution of the Bt isolates according to their nematicidal Cry protein gene content and the habitat from where they were obtained

	Uncultivated fields, trails	Crop	Factory	Farmland	Forest	Garden	Mill	Storehouse	Wetland
*cry5*	2	12	3	2	2	1	18	22	4
*app6*	3	3	0	0	1	0	0	2	0
*cry12*	0	2	1	2	0	0	1	1	0
*cry14*	1	3	3	1	5	0	1	3	2
*cry21*	1	7	0	2	4	1	7	5	0
*cry5* + *app6*	0	1	0	0	1	1	0	2	0
*cry5* + *cry12*	1	0	0	0	0	0	0	0	0
*cry5* + *cry21*	1	5	0	0	1	0	12	3	0
*app6* + *cry12*	0	1	0	0	0	0	0	0	0
*app6* + *cry14*	0	1	0	0	0	0	0	0	0
*app6* + *cry21*	0	0	0	0	0	0	0	1	0
*cry14* + *cry21*	0	0	0	1	0	0	0	1	0
*cry5* + *app6* + *cry21*	0	1	0	0	0	0	0	1	0
*app6* + *cry14* + *cry21*	0	0	0	0	0	1	0	0	0
Total	9	36	7	8	14	4	39	41	6

Combinations of genes coding for nematicidal Cry proteins were observed in collections 1, 2, and 4. Strains combining three genes were scarce as only three strains (two in collection 1, combining *cry5* + *app6* + *cry21*, and 1 in collection 4 combining *app6* + *cry14* + *cry21*) showed this characteristic (Table 2). The combination of two genes was more frequent (especially in collection 1, with 22 strains showing the combination of *cry5* + *cry21*), but not as usual as finding just one gene per strain (Table 2).

### Combinations of nematicidal Cry protein genes with other cry genes

The combinations of *cry* genes coding for nematicidal proteins with *cry1A*, *cry2*, or *cry3* genes were analyzed. Cry1A and Cry2 proteins have been described as toxic for Lepidopteran species and Cry3 for Coleopteran species (van Frankenhuyzen 2009). The results, summarized in Table 4, show that the most frequent combinations were *cry5*, *cry14*, *cry21*, or *cry5* + *cry21*, with *cry1A* + *cry2* genes. But it is worth noting that an important part of the strains harboring *cry5*, *cry14*, *cry21*, or *cry5* + *cry21* were not combined with any of these known family genes.

**Table 4:** Number of strains with nematicidal Cry protein genes which show a combined occurrence of *cry1A*, *cry2*, or *cry3* genes

	*cry1A*	*cry2*	*cry3*	*cry1A* + *cry2*	*cry1A* + *cry3*	*cry2* + *cry3*	*cry1A* + *cry2* + *cry3*	None
*cry5*	7	4	1	38	0	1	1	14
*app6*	2	0	0	2	1	0	1	3
*cry12*	1	1	0	1	1	0	0	3
*cry14*	1	0	0	10	0	0	0	8
*cry21*	2	1	0	14	1	0	0	9
*cry5* + *app6*	0	0	0	1	0	0	0	4
*cry5* + *cry12*	1	0	0	0	0	0	0	0
*cry5* + *cry21*	1	0	0	10	0	0	0	11
*app6* + *cry12*	0	0	0	0	0	0	0	1
*app6* + *cry14*	0	1	0	0	0	0	0	0
*app6* + *cry21*	0	0	0	0	0	0	0	1
*cry14* + *cry21*	0	0	0	0	0	0	0	2
*cry5* + *app6* + *cry21*	0	1	0	0	0	0	0	1
*app6* + *cry14* + *cry21*	0	0	0	1	0	0	0	0
Total	15	8	1	77	3	1	2	57

### Crystal morphology

**Fig. 3:**
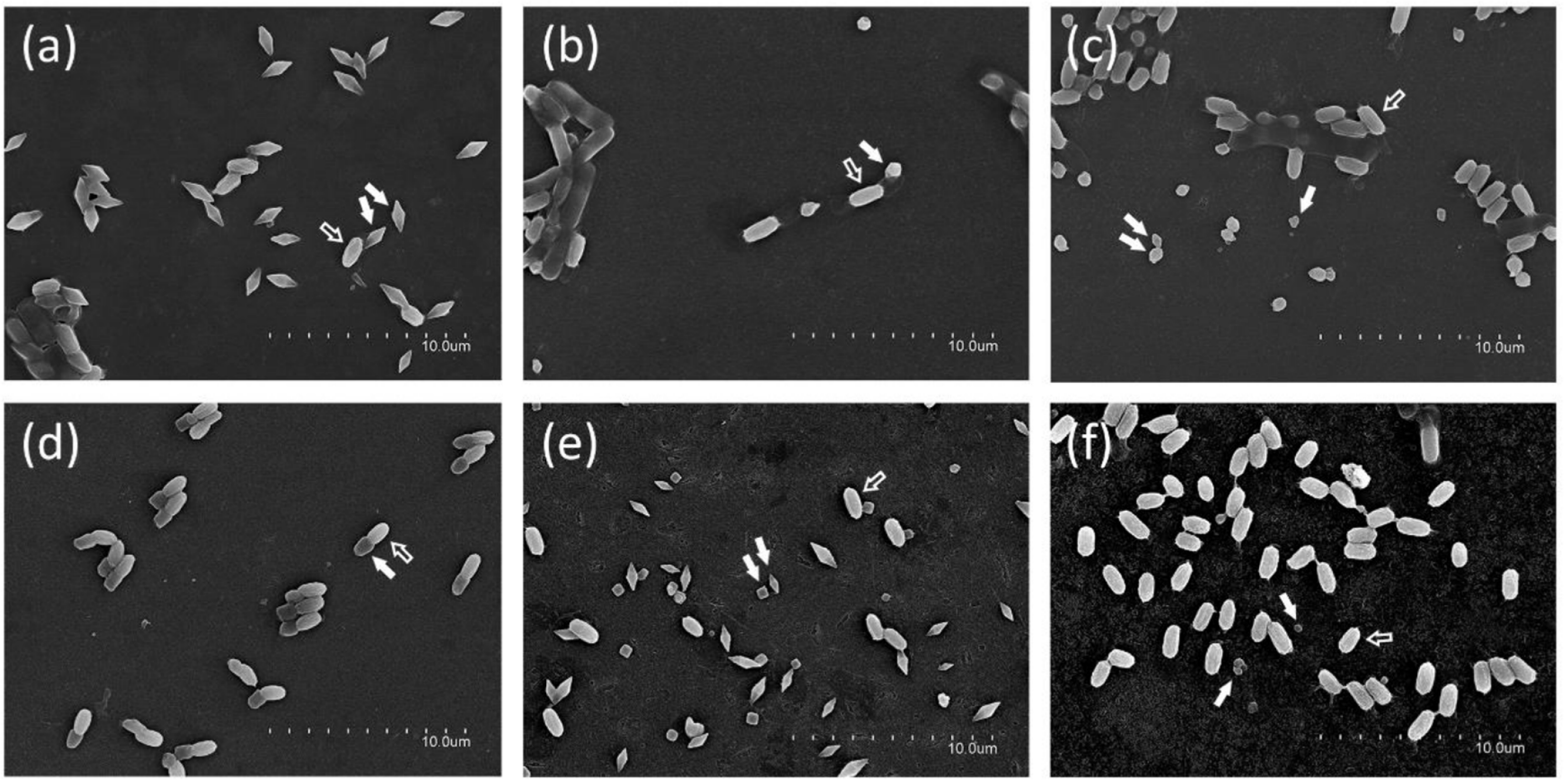
Scanning electron microscopy of Bt strains showing parasporal crystal inclusions with different morphologies. **a** Bipyramidal, **b** round, **c** irregular, **d** adhered to the spore, **e** two-crystals, and **f** small. In the images, white arrows point to crystals and empty arrows point to spores

The correlation between nematicidal Cry protein gene content and crystal shape was also studied (Table 5). The crystal morphologies observed were grouped into 6 different morphological classes: bipyramidal, round, irregular, adhered to the spore, two-crystals, and small. An example of each type of crystal visualized by SEM is shown in Fig. 3. The most frequently observed morphology associated with strains harboring genes coding for nematicidal Cry proteins was the category “small” which consisted of small crystals with different shapes frequently difficult to classify due to the small size but mostly round or elongated. These crystals were detected in strains containing any of the nematicidal Cry protein genes, and also in strains with the combination of *cry5* + *cry21* genes. The following most abundant morphology was “bipyramidal,” and the positive strains with this type of crystals contained frequently *cry5*, *cry14*, or *cry21* genes. The following most abundant crystal shape was “two-crystals,” found mostly in strains carrying the *cry5* gene or the combination *cry5* + *cry12*.

**Table 5:** Classification of the Bt strains according to their crystal shapes and nematicidal Cry protein gene content

	Bipyramidal	Round	Irregular	Adhered to spore	Two-crystals	Small
*cry5*	19	6	5	1	12	23
*app6*	1	0	1	0	0	7
*cry12*	1	0	1	0	1	4
*cry14*	7	3	4	1	3	1
*cry21*	9	1	0	2	5	10
*cry5* + *app6*	2	0	0	0	0	3
*cry5* + *cry12*	0	0	0	0	0	1
*cry5* + *cry21*	3	2	1	0	6	10
*app6* + *cry12*	0	0	0	0	1	0
*app6* + *cry14*	0	0	0	0	1	0
*app6* + *cry21*	0	1	0	0	0	0
*cry14* + *cry21*	0	0	0	1	1	0
*cry5* + *app6* + *cry21*	0	1	0	0	0	1
*app6* + *cry14* + *cry21*	0	0	0	0	1	0
Total	42	14	12	5	31	60

### Expression of the genes coding for nematicidal Cry proteins

The ability of the strains carrying nematicidal Cry protein genes to produce nematicidal proteins was studied through the proteomics analysis of their crystal inclusions. The results are summarized in Table 6, which shows that the number of strains expressing nematicidal Cry proteins can be considered low (23 strains out of 164, about 14%).

**Table 6:** Bt strains harboring and expressing nematicidal Cry protein genes

Nematicidal Cry protein gene/s detected by PCR	Strains harboring the gene/s	Strains expressing the nematicidal protein/s	Nematicidal protein/s expressed	Strains co-expressing Cry1 and Cry2	Strains expressing neither Cry1A nor Cry2 nor Cry3
*cry5*	66	6	Cry5	6	0
*app6*	9	0	–	0	0
*cry12*	7	1	Cry12	0	1
*cry13*	0	0	–	0	0
*cry14*	19	0	–	0	0
*cry21*	27	3	Cry21	1	2
*xpp55*	0	0	–	0	0
*cry5* + *app6*	5	5	0 Cry5, 2 App6, 3 Cry5 + App6A	2	3
*cry5* + *cry12*	1	0	–	0	0
*cry5* + *cry21*	22	6	3 Cry5, 1 Cry21, 2 Cry5 + Cry21	3	3
*app6* + *cry12*	1	0	–	0	0
*app6* + *cry14*	1	0	–	0	0
*app6* + *cry21*	1	0	–	0	0
*cry14* + *cry21*	2	1	Cry21	0	1
*cry5* + *app6* + *cry21*	2	1	Cry5 + App6A	0	1
*app6* + *cry14* + *cry21*	1	0	–	0	0
Total (at least one gene)	164	23		12	11

The *cry5* gene was the most abundant one in the screening, but the Cry5 protein was present in the crystal in just 6 out of 66 strains harboring it (9%). A similar situation was found with the rest of the strains harboring single nematicidal Cry protein genes. Indeed, Cry12 and Cry21 were detected in only 1 and 3 of the 7 and 27 strains that carry *cry12* and *cry21* genes respectively (14% and 11%). None of the strains carrying *app6* or *cry14* expressed them.

The highest correlation between nematicidal Cry protein genes and their expression was observed in the 5 strains carrying *cry5* + *app6* genes. However, we must highlight that just three of the strains expressed both genes, and the other two expressed only *app6*. Regarding the strains with the rest of gene combinations, from the 22 strains carrying *cry5* + *cry21*, just 6 strains have nematicidal proteins in their crystals (27%), and only two of them have both proteins. From the 2 strains harboring *cry14* + *cry21* and the two strains harboring the *cry5* + *app6* + *cry21* combination, only one of each expressed some of their genes.

## Discussion

*Bacillus thuringiensis* (Bt) is a gram-positive bacterium that in the sporulating phase accumulates pesticidal proteins (Cry and Cyt proteins) in a parasporal crystal. So far, seven families of Cry proteins that show toxicity against nematodes have been identified: Cry5, App6 (formerly Cry6), Cry12, Cry13, Cry14, Cry21, and Xpp55 (formerly Cry55) (Jouzani et al. 2017). The aim of this study was to screen (using PCR) four Bt collections stored at the facilities of the Genetics Department, at the University of Valencia seeking for Bt genes coding for proteins active against nematodes. These Bt collections comprise the Bt strains analyzed formerly for different purposes (Iriarte et al. 1998; Hernández-Rodríguez and Ferré 2009; Bel et al. 1997, Hernández et al. 2005) as well as other Bt strains not included in the previous studies.

The PCR screening was performed with some primers described in the bibliography (Ejiofor and Johnson 2002; Bravo et al. 1998), which had been also used by other authors in screening studies looking for *cry5*, *app6*, *cry12*, *cry13*, *cry14*, or *cry21* (Jouzani et al. 2008a, b; Wang et al. 2012; Vidal-Quist et al. 2009). But besides, with primers designed in this work taking into account conserved regions common to the new gene allele sequences described so far, and with primers for the detection of the *xpp55* gene (never included in the previous screening studies).

The screening results showed that there was at least one nematicidal Cry protein gene in 20% of the strains (164 out of 846). But this percentage was not equally distributed among the collections: there was at least one nematicidal Cry protein gene in 33% of the collection 1 strains, in 5% of the collection 3 strains, and in 15% of the strains of collection 2 and collection 4. These numbers, although variable, are in the range of other similar studies. Indeed, Wang et al. (2012) detected *cry5-12–14-21*, *app6*, and *cry13* genes in 8.35% of the strains of a Bt collection obtained from forest samples in China (Wang et al. 2012), and Jouzani et al. (2008a, b) detected at least one nematicidal Cry protein gene belonging to these six families in 31.5% of the strains of an Iranian Bt collection of 70 isolates (Jouzani et al. 2008a). On the other hand, an extensive screening performed with 496 strains obtained from different geographical regions in Mexico that covered different climate zones, failed in detecting strains carrying *cry5*, *cry12*, *cry13*, *cry14*, or *cry21* genes (Bravo et al. 1998), probably due to the usual low frequency of *cry13* genes and to a possible low specificity of the primer pair used for the common detection of *cry5* + *cry12* + *cry14* + *cry21* genes. However, this common primer was used by other authors in a screening of 376 Bt strains isolated from citrus orchards (Vidal-Quist et al. 2009) and in the screening of forest samples in China (Wang et al. 2012) revealing 0.8% and 5.78% of positive strains respectively. Regarding *xpp55*, the present study is the first that includes this gene in the Bt screening. Unfortunately, *xpp55* was not found in any of the strains of the 4 collections despite using two different pairs of primers designed with the more conserved regions of the available gene sequences (Crickmore et al., 2022).

Wang et al. (2012) showed that the number of positive strains found by PCR using the primer pair designed for the common detection of *cry5* + *12* + *14* + *21* was higher than the number of strains harboring *app 6* or *cry13*. However, Jouzani et al. (2008a) found that *app6* was the most frequently found nematicidal Cry protein gene, followed by *cry14*, *cry21*, and *cry5*. In the present study, *cry5* was the most abundant gene in collections 1, 2, and 3, similarly to Wang et al. (2012). But this pattern was not repeated in collection 4 despite sharing geographic areas and habitats with collection 1. In collection 4 (with 15% of strains carrying at least one nematicidal Cry protein gene in contrast to 33% in collection 1), the most frequent gene was *cry14* followed by *cry5*. These results suggest that the abundance of nematicidal Cry protein genes can be variable, regardless of the time and the location of sampling. Taking all the data from the four collections together, *cry5* (alone or in combination with other nematicidal Cry protein genes) was the most abundant gene, found in 11.3% of the strains, followed by *cry21* (in 6.5% of the strains). Indeed, the statistical analyses pointed out that these two genes were genetically linked. Regarding the *app6* gene, no amplicon was obtained when using the Cry6F/Cry6R primers (specific for *app6B* allele) but the *app6A* gene was found in 2.3% of the Bt isolates. Bt strains harboring *cry14* or *cry12* genes were also found in 2.7% and 1% of the strains, respectively. There were no strains positive for the *cry13* or *xpp55* genes.

In screening publications assessing the abundance of Bt genes in diverse geographical regions and habitats, *cry1* genes appear as the most abundant, and *cry2* or the combinations of *cry1* + *cry2* alone or with other genes are also highly frequent (Djenane et al. 2017, Lone et al. 2017, Sauka and Benintende 2017, Boonmee et al. 2019, Vidal-Quist et al. 2009, Bravo et al. 1998, Yılmaz et al. 2017, Jain et al. 2017, Wang et al. 2003, Porcar and Juarez-Perez 2003). Then, it was not surprising to find a high frequency of combinations of the genes coding for Bt Cry nematicidal proteins with *cry1A*, *cry2*, or with *cry1A* + *cry2* genes, being the combination with the last grouping the most frequent one (Table 4), including the strains harboring *cry5* + *cry21* genes. Also, it was not surprising that only one of the strains harboring nematicidal protein genes carried *cry3*, as the frequency of this gene, when included in Bt screening studies, resulted to be very low or even zero (Hongyu et al. 2000, Vidal-Quist et al. 2009, Hernández et al. 2005, Lone et al. 2017, Bravo et al. 1998, Ben Dov et al. 1997).

In general, the nematicidal Cry protein genes were most frequently found in dust samples from storehouses or mills followed by crop soils, and in lower frequency, in samples from other habitats such as wetlands, uncultured fields and trials, forests, farms, factories, and gardens. This is in agreement with what has been described in other screening studies where a high frequency in the recovery of Bt strains (accompanied by higher variability in the type of Bt strains found) was reached in dust samples from storehouses, silos, or mills (Iriarte et al. 1998, Hernández-Rodríguez and Ferré 2009, Sauka and Benintende 2017, Wang et al. 2003).

The parasporal crystal shape in the Bt strains harboring nematicidal Cry protein genes was studied. Six morphological classes were established (bipyramidal, round, irregular, adhered to the spore, two-crystals and small). The crystal shape “small” was the most frequent in Bt strains carrying genes encoding Cry nematicidal proteins (37%). Indeed, this was the morphology most associated with Bt strains harboring *cry5*, *app6*, *cry12*, *cry 21*, or *cry5* + *cry21* genes. Differently, the bipyramidal shape was the most frequent in strains harboring the *cry14* gene. The strain YBT-1518 that harbored *xpp55Aa1*, *app6Aa2*, and *cry5Ba2* genes (but only expressed *xpp55Aa1* and *app6Aa2*) had a “rice-shaped” crystal (Guo et al. 2008). When the genes were cloned and expressed individually, the strains expressing *xpp55Aa1* or *app6Aa2* formed rice-shaped crystals, but the one expressing *cry5* produced bipyramidal crystals (Guo et al. 2008). Bipyramidal crystals and two-crystals were also found often in strains with the *cry5* gene in the present study. The study of 22 nematode-active cry gene containing isolates performed by Jouzani et al. (2008a) found that about 50% of the isolates contained more than one crystal, whereas in the current study, this percentage is much lower (19%). The variety in crystal morphologies and apparent lack of recognizable correlation between the gene content and crystal shape, also observed by Jouzani et al. (2008a), is most probably due to the different expression rates of the genes coding for Cry nematicidal proteins in the strains and to the possible combination of them with other Cry proteins that could be also expressed.

The PCR analysis has been considered a powerful tool to identify specific *cry* genes, to characterize numerous Bt isolates, and to predict their biological activity (Carozzi et al. 1991, Ben Dov et al. 1997). But it is not an accurate indicator of toxicity as genes could be truncated, mutated, or not expressed (Guo et al. 2008; Ferrandis et al. 1999; Sajid et al. 2018; Masson et al. 1998; Khorramnejad et al. 2018; Porcar and Juarez-Perez 2003). Toxicity screenings are the most used approach to know if the PCR screened strains are toxic or not to the target pest species (as examples, see Jouzani et al. 2008a; Wang et al. 2012; Boonmee et al. 2019; Hongyu et al. 2000), although it is an expensive and time cost methodology. In this work, to assess the nematicidal capacity of the strains carrying nematicidal Cry protein genes, we have analyzed directly the protein expression by LC/MS–MS analysis. The results obtained show that the nematicidal Cry protein genes are not expressed widely as only 14% of the strains present nematicidal Cry proteins in their parasporal crystals.

None of the strains harboring *app6* or *cry14* genes expressed them. In addition, only 6 out of the 66 strains carrying *cry5* (9%) expressed the Cry5 protein. And Cry21 and Cry12 were found in 11% and 14% of the strains that carry their respective genes. Regarding the strains harboring nematicidal *cry* gene combinations, the combination of *cry5* + *app6* seemed to be the best in terms of expression (combined or not with *cry1A* and *cry2* expression), although not all the strains expressed both genes. Similarly, Guo et al. (2008) that described the strain YBT-1518 which harbored the genes *xpp55Aa1*, *app6Aa2*, and *cry5Ba2* observed that *xpp55* and *app6* which were in the same plasmid were transcribed, whereas cry5Ba2, although complete in the strain, remained cryptic.

The use of PCR coupled with LC/MS–MS analyses can mean a great reduction of time, resources, and effort when looking for strains with toxicity against specific nematicidal species, as the proteomic analyses allow to discard uninteresting strains and helps to predict the possible nematicidal activity of the interesting ones. For example, all the strains expressing nematicidal proteins in the current work (which produce Cry5, App6, Cry12, or Cry21 proteins) could be toxic for plant parasitic nematodes (Li et al. 2007, 2008; Guo et al. 2008, 2022; Wang et al. 2012; Yu et al. 2015; Verduzco-Rosas et al. 2021), and the strains producing Cry5 or Cry21 could be toxic for mammalian intestinal nematodes (Wei et al. 2003, Kotze et al. 2005, Capello et al. 2006, Urban et al. 2013), or could be active against several free-living nematodes (Wei et al. 2003). In addition, it must be taken into account the possible synergistic effects that can occur when combining the toxic action of two or more of these proteins (e.g., App6Aa and Xpp55Aa or Cry5Ca and Cry5Da against *Meloidogyne incognita* (Peng et al. 2011 and Geng et al. 2017, respectively). Taken together, our results show that combining the genetic screening (PCR) with LC–MS/MS for crystal protein analysis results in a complementary and powerful way to assess the potential of Bt isolates, for gene assessment and for toxicity specificities.

In summary, in this work, we have analyzed by PCR a large and diverse number of Bt strains (subdivided into four collections) for their content in genes that encode proteins from the seven Bt Cry nematicidal protein families. In general, the nematicidal Cry protein genes were found with relatively high frequency (not correlated with the habitat, with the presence of other pesticidal Cry protein genes or with the shape of the crystal), but the genes were expressed in a low percentage of the positive strains, highlighting the great potential of the latter as possible nematode control agents.

## Data Availability

The Bt strains and the datasets generated and/or analyzed in the current study are not publicly available due to being part of a European project (EcoStack, Grant Agreement no. 773554) whose results may be patentable, but can be available from the corresponding author under a confidentiality agreement signature.
